# Low-dose-rate brachytherapy for patients with transurethral resection before implantation in prostate cancer. Long-term results

**DOI:** 10.1590/S1677-5538.IBJU.2014.0531

**Published:** 2016

**Authors:** Pedro J. Prada, Javier Anchuelo, Ana García Blanco, Gema Payá, Juan Cardenal, Enrique Acuña, María Ferri, Andrés Vázquez, Maite Pacheco, Jesica Sanchez

**Affiliations:** 1 Department of Radiation Oncology, Hospital Universitario Marqués de Valdecilla, Santander, Cantabria, Spain;; 2 Department of Radiation Physics, Hospital Universitario Marqués de Valdecilla, Santander, Cantabria, Spain

**Keywords:** Brachytherapy, Transurethral Resection of Prostate, Prostatic Neoplasms

## Abstract

**Objectives:**

We analyzed the long-term oncologic outcome for patients with prostate cancer and transurethral resection who were treated using low-dose-rate (LDR) prostate brachytherapy.

**Methods and Materials:**

From January 2001 to December 2005, 57 consecutive patients were treated with clinically localized prostate cancer. No patients received external beam radiation. All of them underwent LDR prostate brachytherapy. Biochemical failure was defined according to the “Phoenix consensus”. Patients were stratified as low and intermediate risk based on The Memorial Sloan Kettering group definition.

**Results:**

The median follow-up time for these 57 patients was 104 months. The overall survival according to Kaplan-Meier estimates was 88% (±6%) at 5 years and 77% (±6%) at 12 years. The 5 and 10 years for failure in tumour-free survival (TFS) was 96% and respectively (±2%), whereas for biochemical control was 94% and respectively (±3%) at 5 and 10 years, 98% (±1%) of patients being free of local recurrence. A patient reported incontinence after treatment (1.7%). The chronic genitourinary complains grade I were 7% and grade II, 10%. At six months 94% of patients reported no change in bowel function.

**Conclusions:**

The excellent long-term results and low morbidity presented, as well as the many advantages of prostate brachytherapy over other treatments, demonstrates that brachytherapy is an effective treatment for patients with transurethral resection and clinical organ-confined prostate cancer.

## INTRODUCTION

Brachytherapy has rapidly gained popularity as an accepted, effective and safe therapy for localized prostate cancer. There are robust follow-up data beyond 10 years that show similar biochemical control rates to radical prostatectomy and external beam radiotherapy (1-3).

Many patients with preexisting lower urinary tract symptoms have been considered poor candidates for seed implants; however there have been few rigorous studies of the contraindications for brachytherapy. Several authors (4, 5) reported a higher risk of post-implant urinary incontinence in patients with a prior transurethral prostate resection (TURP).

Transurethral prostate resection, developed in 1930, is a surgical procedure which consists on removal of the prostate parenchyma proximal to the verumontanum and distal to the bladder neck as a treatment for urinary obstruction. It is done without penetrating the prostatic capsule. The incontinence rate from TURP alone is low, ranging from 1% to 5% (6).

There has been little research on the safety and effectiveness of low dose rate brachytherapy performed in patients with prior TURP. The objective of the present study was to report the clinical outcome, side-effects and complications after permanent implantation of 125 I seeds for early prostate cancer in patients with a prior TURP with up to 10 years of follow-up.

## MATERIALS AND METHODS

### Selection of patients

In all, 57 patients with a TURP prior to brachytherapy were treated between January 2001 and December 2005; the median (range) follow-up was 104 (11–154) months. Patients were staged according to the American Joint Committee on Cancer 6^th^ edition clinical staging guidelines (7) using a directed history, physical examination and TRUS. All patients had their serum PSA level measured and Gleason score histological grading. The tumor characteristics are shown in Table-1.

**Table 1:** Patient and tumor characteristics (n=57).

Characteristics	Nº Patients (%)
**Stage**	
≤T2a	50 (88%)
T2b	7 (12%)
**Gleason score**	
≤6	48 (84%)
=7	8 (14%)
>7	1 (2%)
**Pretreatment PSA (ng/mL)**	
≤10	41 (72%)
10.1-20	15 (26%)
>20	1 (2%)
Mean: 9/Median 8 (1.4-47)	
**Adjuvant hormonal ablation**	
Yes	23 (40%)
No	34 (60%)
**Age at diagnosis (year)**	
≤60	12 (12%)
61-70	25 (44%)
>70	25 (44%)
**Risk Level**	
Low Risk	48 (84%)
Intermediate Risk	8 (14%)
High Risk by PSA	1 (2%)
Gland Vol. Implant (cc): Mean: 36/Median 35 (12-66)	

In all patients TURP was done some months before brachytherapy (mean 70 months, range 4-132 months). All patients underwent small or medium not large TURP. The mean prostate volume, as measured by ultrasound before brachytherapy was 36cc (range 12-66cc). The mean resected volume was small (15g). The resected volume was noted at the time of brachytherapy but did not cause any technical problem to the seed implant to get enough tissue (>1cm) at TURP level.

### Definition groups

The Memorial Sloan Kettering group definition (8) was used to classify patients into risk groups; low-risk patients were T1c or T2a, with a PSA level of ≤10ng/mL and Gleason score ≤6; intermediate risk was T2b, PSA level 11–20ng/mL or Gleason score ≤7; and high risk was ≥T2c, PSA level>20ng/mL or Gleason score >7, or two intermediate-risk criteria.

### Hormonal Therapy

In our patient population, 40% received hormone therapy before brachytherapy; this treatment was prescribed by the urologist, waiting for the definitive brachytherapy treatment. Hormonal therapy was given for 3 months and then stopped. The mean prostatic volume at implantation was 36 (12-66 cc).

### Treatment

All patients received brachytherapy alone with I-125. The prescription dose was 145 Gy to the reference isodose (100%) according to the TG-43 (9). The target volume of the implant was the prostate gland plus a 2-5mm peri-prostatic area.

The technique used in the implantation was based on intra-operative planning with real-time dynamic dose calculation with peripheral loading. The implantation technique has been previously described (10, 11).

To decrease rectal toxicity, transperineal hyaluronic acid injection into the peri-rectal fat was used to consistently displace the rectal wall away from the radiation sources in 6 patients. We considered that the increase in distance (mean 2cm along the length of the prostate) would be enough to provide a significant reduction in radiation dose from LDR brachytherapy (12).

Patients were followed with symptom assessment and PSA determinations every 3 months for the first year, every 6 months for the second year and yearly thereafter.

### Toxicity

Morbidity was reported according to the Common Terminology Criteria for Adverse Events (CTCAE 4.0). Toxicity and sexual side effects was scored by the physician.

### Statistical considerations

Distant metastases disease was defined by an imaging study or physical examination that demonstrated cancer outside of the prostate and its regional nodes. Failure in tumor-free survival (TFS) analyses was represented as detection of local and/or systemic tumor relapse. Biochemical failure was defined according to the “Phoenix definition” (13) consensus panel statement. Estimated likelihood of events was calculated by the Kaplan-Meier method from the time of completion of brachytherapy procedure. The statistical significance of the difference between estimated event-free curves was calculated with the Log Rank test. Multivariate analysis was performed using the Cox proportional hazards model (14).

## RESULTS

For the entire cohort of 57 patients, 3 had evidence of biochemical relapse, 2 had a clinical relapse and 1 died from prostate cancer; 6 patients died of other illnesses.

**Figure 1:**
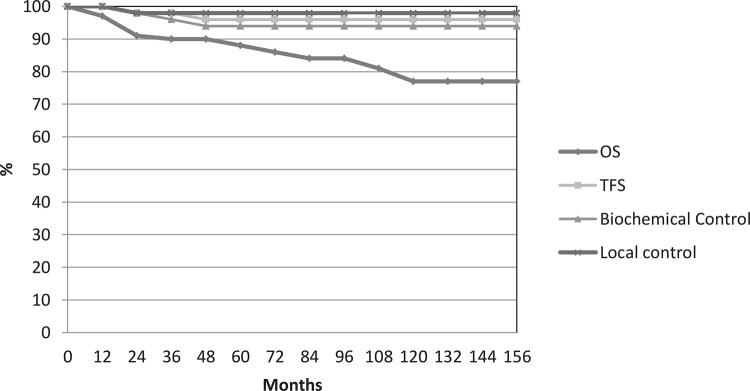
Actuarial analysis of all 57 patients for overall survival, tumor-free survival and biochemical control and local control.

The overall survival according to Kaplan-Meier estimates was 88% (±6%) at 5 years and 77% (±6%) at 12 years. The 5 and 10 years for failure in tumor-free survival (TFS) were 96% and 96% (±2%) respectively, whereas for biochemical control was 94% and (±3%) at 5 and 10 years respectively, 98% (±1%) of patients being free of local recurrence (Figure-1).

Cox proportional-hazards regression revealed no statistical significant association for clinical T stage, Gleason score, pretreatment PSA, age, brachytherapy dose (D90), hormonal ablative treatment and biochemical failure.

The actuarial biochemical control with Gleason score was 95% and 89% for patients with Gleason score of ≤6 and 7, respectively (P=0.4). The correlation with pretreatment PSA the biochemical control was 97% and 89% for patients with PSA of ≤10 and >10ng/ml, respectively (P=0.26).

T stage was not significant (P=0.38) for biochemical control (100% for ≤T2a and 93% for T2b).

Mean patient age was 69 years (range 55-77). The actuarial analysis of biochemical control at ages less than 61, 61 to 70 and greater than 70 years demonstrated no significant difference, as younger and older patients benefited equally (P=0.26).

The actuarial biochemical control was the same 93% (P=0.37), in patients who received hormones and in those who did not.

Increasing the dose received by 90% of the prostate volume (D90) from ≤160 Gy and >160 Gy was not associated with improved biochemical control (P= 0.37).

All 57 patients were discharge from the center the same day of the procedure between 6-8 hours of implantation. All patients have been seen in follow-up and the CTCAE toxicity criteria were utilized to score acute and late complications.

### Acute and Chronic Urinary Toxicity

Moderate increase in urinary irritation (urethritis) occurred in the third month after treatment; the acute GU grade II toxicity was 9%.

The incontinence rate prior to brachytherapy grade I were 9% (5 patients) and grade II, 3% (2 patients). Only a patient without prior incontinence reported incontinence after brachytherapy (1.7%). Acute urinary retention was seen in 1 (1.7%) patients, requiring a temporary post-implant bladder catheter. Late urinary retention occurring more than two year after treatment was reported in 1 (1.7%) patients.

The chronic genitourinary complains grade I were 7% and grade II, 10%. A patient had late urethral stricture, requiring urethral dilations.

### Lower Gastrointestinal Toxicity

At six months 94% of patients reported no change in bowel function.

The incidence of rectal ulceration and/or recto-urethral fistula (Toxicity grade III-IV) has been observed in 2 patients (3.5%) after rectal biopsy.

Intermittent rectal bleeding was reported in 3 patients (5%). In 6 patients (11%) transperineal hyaluronic acid injection into the peri-rectal fat was used to consistently displace the rectal wall away from the radiation sources; no mucosal damage and no macroscopic rectal bleeding were observed in this group.

No patients with perineal pain were reported.

### Sexual function

Of the 17 (30%) patients who were potent preoperatively, 82% were potent postoperatively. Potency was defined as the ability to achieve an erection that was sufficient for intercourse.

## DISCUSSION

Our encouraging results are in concordance with the experience of other institutions (15-19). Multivariate and univariate analyses show that the pretreatment PSA level, Gleason score and T stage were not a significant variable for biochemical control. In the present series, hormonal ablative treatment was given for 3-4 months and did not improve biochemical control.

In our series the incontinence grade 1 rate prior to brachytherapy was 9% (5 patients) and grade II, 3% (2 patients) but incontinence chronic toxicity TURP-related after brachytherapy was reported only in a patient (1.7%). Late urethral stricture was reported in 1 (1.7%) patients.

Moran et al. (20) analyzed 171 patients with T1a-T1b prostate cancer who underwent prior TURP. The mean urinary function and bother score for the entire study group was 83.5±19.5 and 82.5±23.7, respectively. Multivariate analysis revealed higher pretreatment International Prostate Symptom Scores to have significant negative impact on urinary function and bother scores. They concluded that it is feasible LDR brachytherapy in selected patients with prior TURP, with low impact on urinary function and bother scores.

Wallner et al. (21) in 19 patients reported a 6% incontinence rate in a TURP patient group.

Stone et al. (22) suggest that brachytherapy can be safely performed with a low risk of urinary incontinence if a real-time method combined with peripheral loading is used, but they point out that it could result in a higher risk of urinary incontinence.

Ramirez et al. evaluated urinary incontinence in 16 patients with prior TURP and find lower urinary tract symptoms or urinary incontinence after an average of 30 months (23).

Cesaretti et al. (24) evaluated prostate brachytherapy dosimetry outcomes relative to the transurethral resection of the prostate in 73 patients and they concluded that a visible residual TURP cavity (≥10% of a prostate volume) did not appear to be a statistically significant hindrance to proper dosimetric outcome.

Salembier C et al. (25) evaluated prospectively in a multicenter setting the ability of centers to perform pre-implant permanent prostate brachytherapy planning with dosimetric goals and constraints based on the Groupe de Curiethèrapie-European Society for Radiotherapy and Oncology guidelines in patients with prior TURP concluding that it is feasible.

Brachytherapy for patients with a prior TURP and early-stage prostate cancer is effective, with long-term biochemical freedom from recurrence independently of the age of the patients (as younger and older patients benefited equally). The present study showed low toxicity when the dose to any segment of the TURP defects is limited to ≤100% of the prescription dose and the actuarial biochemical control was excellent (95% for patients with Gleason score of ≤6). The median hospital stay for our patients was 12 h (6-8) h after implantation; there are no other alternative treatments with a shorter hospital stay.

The present complications rates were in accordance with the experience of other institutions using permanent implants of 125 I (19-24) for patients with prior TURP.

In conclusion, with the present long-term data, using intra-operative planning with real-time dynamic dose calculation with peripheral loading, LDR brachytherapy provides excellent biochemical control rates for patients with localized prostate cancer and prior TURP, and low urinary and gastrointestinal morbidity.
